# Resources and transversal competencies to reconcile child and parental responsibilities: A mini-review of the impact of COVID-19 on the Italian sandwich generation

**DOI:** 10.34172/hpp.2023.21

**Published:** 2023-09-11

**Authors:** Aurelia De Lorenzo, Lynda S. Lattke, Elga Zedda, Emanuela Rabaglietti

**Affiliations:** ^1^SE-CREA Research Group, Department of Psychology, University of Turin, Turin, Italy; ^2^Department of Psychology, University of Turin, Turin, Italy

**Keywords:** Coping strategies, Gender-based differences, Inter-generational dynamics, Sandwich generation, Transversal skills

## Abstract

Longer life spans and the delayed economic and psychological independence of children mean that middle-aged parents find themselves caring for parents and children at the same period in their lives, at times this care may extend to grandchildren and grandparents. The type of care varies depending on each person’s need but also on the gender of the individual who looks after their parents while taking care of their own children. Cultural factors can also come into play. Complications in the lives of those who are part of the Sandwich Generation (SG) may arise affecting the quality of their time, their psyche, physical and financial situation. Having the necessary skills and strategies to cope with these complications is extremely important.

## Introduction

 The term sandwich generation (SG) is commonly used to refer to the middle-aged adult population that cares for their children and, at the same time, for their aging parents.^1,2^ While in the early 1990s the phenomenon of the SG included adult individuals, who found themselves sandwiched between the responsibilities of caring for young children and parents in need of support,^1,2^ today the situation has shifted further. Today, we can speak of a new generation that we might call a double sandwich: the 45-55 age group is indeed caring for young children and/or adolescents and aging parents, but the 55-75 age group includes individuals whose parents are still alive, albeit very old, and whose children, now adults, need assistance in caring for their grandchildren.^3^

 In general, studies, albeit from different perspectives,^4^ agree that the SG consists of adult individuals who due to their age and family relationships are *in the middle* and *squeezed* between young and old, resulting in simultaneous responsibilities and satisfaction of demands towards these ties.^2,5,6^ Hence, both men and women belong to this generation.^5^ The literature confirms, however, the existence of gender differences where middle-aged female populations are more involved in providing support and assistance to both generations.^7-9^ In this regard, we can consider two factors that explain this phenomenon: first, working sandwich generation women experience different challenges related to the multiple, often traditional roles they play at home and in the workplace.^8^ Second, the type of care offered differs at the gender level, a situation that reports differences between them making, at times, the care of SG women heavier still.^10^ With these initial premises about the *middle* generation in mind, the following sections will argue the most salient socio-demographic aspects, possible implications of protective and risk factors at the individual and societal levels, and possible strategies for coping with the phenomenon.

## Inter-generational dynamics: factors to be considered

 Children and parents establish lifelong social relationships, as familial relationships are crucial for the well-being of both generations.^11,12^ In Italy, initial family ties are generally strengthened over time: the generational pact illustrates how parents help their adult children, even when they start their own independent families, and in return receive care and support for their children when they need it. Therefore, even if they have left their family unit, working mothers can count on the support of older relatives such as parents or in-laws.^13^

 When an older person is in good health, this caregiving relationship can lead to personal growth and promote the development of new skills, including less stress,^14^ developing intergenerational relationships between children and older people who experience greater satisfaction from caring for loved ones.^15,16^ It can also strengthen their kinship and family identity.^17^ However, when a parent’s autonomy (whether physical, mental, or emotional) begins to wane, the adult child is often unprepared for the lifestyle changes, almost suddenly becoming part of the SG and find themselves in a situation which means having to offer double care^18^ while being parents to their own children but in some cases also parents to their own parents.^19,20^

 To understand these changes, it is important to consider the backdrop in which they happen: The Western world has declining birth rates, adults become parents later in life, and people generally live longer thanks to greater awareness of their own care and medical technologies that help with early detection of disease.^2,21-23^ Some of the consequences of this backdrop are that children stay at home with their parents longer than usual, which means that economic independence is significantly delayed^17,24,25^; so that middle-aged adults find themselves caring for their elderly parents, who unexpectedly have less autonomy while caring for their adolescent children. When families move away from their parents’ homes and live in different cities, regions, or countries, this distance can exacerbate this situation.^18^ When one or more parents in a couple show signs of illness, they must alternate between how to care for their vulnerable parents while caring for their children in cities where they themselves may not have an established network, having moved from another city or country.^26^ This may include negotiating transnational care with siblings or alone if the SG adult is an only child.^27^ In the case of countries such as Italy, where birth rates are lower, this means that fewer children share the responsibility of caring for their elderly parents.^17^ In addition, depending on the culture of origin, feelings of guilt may come into play for not being closer,^8,27^ and creative ways must be found to provide emotional care from a distance.^28^ The decreased autonomy of older parents can lead to a drastic change in roles and perspectives on life, a rescaling of time (i.e.: less time to oneself) with the added dimension of having to accept the psychological, social-relational, emotional, and mental changes that older parents go through along with caring for their well-being.^25^

 The complexity of the parenting role is defined by family systems theory, which states that parents must participate in and respond to the care of multiple family subsystems, such as individual-level self-care, coordination of co-parenting demands, and parenting needs.^29^ In general, the nature of the parent-child relationship changes frequently: children grow up, parents grow older, and adult children take on a caregiving role for older parents.^30^ Thus, the support and tensions that arise from intergenerational bonds during stressful times between family roles are critical for middle-aged adults.^8^ This situation was highlighted by Covid-19, which created greater difficulties in managing the boundaries between family and work^31^ in the context of economic concerns about reduced working hours or job loss. This is due to the fact that, despite the presence of a partner, caregiving and support tasks were mainly delegated to women during the pandemic period, even if they were engaged in smart work.^32,33^ After the lockdown, first men and later women returned to work outside the home, often choosing to leave their jobs to meet family needs.^34,35^

 In this scenario of expecting and providing care, it can be challenging to balance work, caregiving, and family life in general. According to an Italian study, 35.9% of mothers and 34.6% of fathers complained about the unresolved difficulties of their work schedules and commuting, which led to time constraints in providing such care.^20^ In trying to juggle these obstacles and meet multiple needs, SG individuals often experience guilt and frustration.^28^ This is exacerbated when the social expectation has been made official through government policy, as in China.^36^ When the children of SG individuals are under the age of 15, they may face greater stress that affects their mental health.^2,37^ Not only is leisure time dramatically reduced, but overall ‘time management’ becomes more difficult,^17^ often leading SG individuals, especially women, to consider quitting their jobs^21^ because they burn out or need to reduce their work hours to provide care at home.^33^ In this process of reorganization and readjustment, “*family roles and responsibilities are transformed*”^17,21^ (p.287), and gender tensions may arise, especially when priority is given to men and their work as the main breadwinners of the family,^17^ leading to different roles within the family and how each SG person manages their time.

## Caring and gender association

 Jang et al^18^ note that in all cultures, some form of care is expected from one’s children when the time comes. However, the degree of involvement in this care may vary across cultures.^38^ In Northern European and American cultures, the tendency is for older parents to maintain their independence as much as possible, whereas, in Southern European countries, such as Italy or Latin American countries, the tendency is for SG adults to care for their parents and thus be close by Baldassar et al.^26^ As globalization increases, caregiving and the importance of caregiving varies depending on the geographic distance between the SG adults and their parents, the type of relationship they have,^26^ and who is caring for the person.^38^ Vlachantoni and colleagues^39^ point to the UK context where the needs of older people “*...can be met through a combination of contributions from the informal, formal state, and formal paid sectors*” (p. 321). Nevertheless, they note that the informal sector of family and friends remains predominant, as it is in most countries around the world.

 While caregiving is directly provided by one’s children, the literature confirms the existence of gender differences, particularly among the middle-aged female population, as they are more involved in supporting and helping both generations.^7-9,18^ Traditionally, women tend to provide more emotional support and engagement.^10,33,40^ Working women of the sandwich generation face different challenges related to the multiple and traditional roles they play at home and at work,^8,41,42^ including societal and cultural expectations.^10,26^ According to Evans and colleagues,^8^ women perform worse in this situation and in the use of individual strategies compared to men in the sandwich generation with the same combination of roles (family caregiving and work), even in relatively egalitarian societies.^10^ Negative consequences at work include higher absenteeism due to stressful family situations, leaving work early, and emotional stress that affects work performance.^43^ In general, stress levels increase when individuals who belong to the sandwich generation have to deal with the demands of their children and aging parents at the same time, which limits the time they have for their personal lives,^44^ such as healthy behaviors for themselves (exercise or healthy eating).^45^ Multigenerational caregiving may influence health behaviors because of the reduced relevance of personal goals, as it increases the tendency to worry about meeting the needs of others by increasing personal stress.^46^ Any form of continuous caregiving, which is often invisible and unrecognized by society,^47^ can increase stress, mental exhaustion, and symptoms of anxiety and depression.^48-50^

 In men, on the other hand, practical caring predominates over both genders.^10^ Gender differences in work concern both individual and family characteristics. At the European level, for example, Italy ranks third after Greece and Romania in gender differences in time spent on family work.^22^ In addition, the type of care provided by men and women may help explain the direction of that care. In a Finnish study, four variables and their directional prevalence were considered: for adolescent children, there was more financial support, caring, and emotional closeness, while for elderly parents, practical care predominated. While this directional prevalence was more or less the same for both genders, women were found to be emotionally closer to their children and provided more care for both groups, elderly parents and children, which can be emotionally stressful.^19^

## Transversal competencies and strategies to cope during this period

 Fostering individuals’ resources related to their transversal skills involves promoting and creating spaces where individuals can develop strategies to promote roles through communication and flexibility of family members,^44^ while balancing them with self-management, social support, and participation in self-care activities.^8^ Some skills that emerge relate to coping through emotion regulation,^18,51^ including taking responsibility and self-control, as well as organization and time management, which are critical to the well-being of both parents and their children.^52,53^ Research on social support, understood as an interpersonal skill, reflects social-emotional competence related to building stable, enduring, and positive relationships with others.^44^

 Indeed, caregivers play a key role in promoting the development of emotion regulation, understood as social-emotional competence, through emotional support of family members.^54^ Interventions that provide support and stress reduction include teamwork, promoting open communication, and encouraging caregiver self-care.^55^ This can be ensured at the system level through counseling services that help family members understand and support parents.^56^

 Resilience is an important resource that can counteract high levels of psychological distress when faced with situations of uncertainty, such as those that occurred during the COVID-19 pandemic, which made the tension between parallel care needs even more complex. COVID-19 often hindered SG individuals’ ability to draw on the resources necessary to cope with the challenges associated with caregiver stress.^57^ Some authors have emphasized the importance of resilience and mutual aid in improving the mental health of family members.^58^ When families become resilient, family members’ risk and vulnerability decrease, which increases their ability to cope with future challenges.^59^ It is critical to incentivize the resources that individuals may have, both organizationally and instrumentally (availability of family supportive organizational practices) and emotionally (from family members or friends).^44,60^ The availability of these resources allows individuals to use them flexibly as the caregiving burden increases.^61^ It is important to focus on individual strategies that allow for a balance of roles that each family member, especially women, share, which in the case of the sandwich generation is complex, multidimensional, and interconnected.^13^

 Often, the negative consequences of being part of the sandwich generation are mitigated by improving one’s physical health through healthy habits.^62^ This includes leisure time,^63^ which is linked to organizational capacity, understood as a cross-cutting competency. In general, a holistic approach is essential for rebalancing the demands and resources of the sandwich generation in both family and work contexts.

## Conclusion

 The SG individuals drive the world’s economy while caring for future adults as well as those who raised them.^2^ Given the complexity of the phenomenon, continued attention from various sectors of society is needed to develop strategies to meet the needs of this multigenerational target group. In addition, research suggests that the development of transversal skills may help in coping by improving the psychological well-being and quality of life of family members.^64^ Developing a gender-sensitive approach to the sandwich generation means taking into account the challenges women face by promoting a balanced distribution of responsibilities between the sexes.^8^ At the system level, it is important to provide support and resources to SG individuals, such as care services for children and the elderly, training programs, and programs to develop transversal skills, i.e., social policies and measures to support families.^20^ To counteract the possible negative effects of the phenomenon, it is necessary to incentivize welfare initiatives aimed at providing useful services to both caregivers and the elderly, including research and financial support through family assistance and psychological support through appropriate information channels.^43^ This could promote the well-being and quality of life of SG individuals by improving and strengthening work-life balance. Only through a comprehensive and integrated approach can we hope to successfully address the problems of the sandwich generation and improve their quality of life.

 At the employers’ level, people who work in an environment that protects the psychological and physical well-being of the person, for example, through leave, family leave, and work-life balance projects, suffer less from being *crushed* by the needs of the family environment.^37^ According to Brenna,^20^ “*...greater flexibility in work schedules, provision of time-limited work leave to prevent job abandonment during years of intense family needs (preschool-age children, parents nearing the end of life), an increase in long-term care services, especially home care services that encourage aging in place, and financial support for child care are all measures that can help reduce the excessive burden on women living in a sandwich*” (p. 422). In addition, employers should also consider alternative strategies, such as yoga and meditation break in the office, as practiced in India, which have been shown to help reduce stress.^65^ On a more individual level, self-care is essential to reduce the emotional exhaustion caused by caring for parents and children.^18^ Part of this self-care involves verbalizing the need for time for oneself and extending family care.^38^

 Last but not least, it is important to promote active aging so that older people remain mentally and socially engaged, as well as physically fit,^21^ and are perceived as individuals who have much to offer not only within the family but also to society.

## Competing Interests

 Not applicable.

## Ethical Approval

 Not applicable.

## Funding

 No funding was provided for the research and writing of this review.

## References

[1] Grundy E, Henretta JC (2006). Between elderly parents and adult children: a new look at the intergenerational care provided by the ‘sandwich generation’. Ageing Soc.

[2] DeRigne L, Ferrante S (2012). The sandwich generation: a review of the literature. Fla Public Health Rev.

[3] La «generazione sandwich» ora riguarda anche i nonni [Internet]. Centro Studi 50&Più. Available from: http://www.centrostudi.50epiu.it/Schede/la_generazione_Sandwich_ora_riguarda_anche_i_nonni. Accessed June 24, 2023.

[4] de Lima EE, Tomás MC, Queiroz BL (2015). The sandwich generation in Brazil: demographic determinants and implications. Rev Latinoam Poblac.

[5] Parker K, Patten E. The Sandwich Generation: Rising Financial Burdens for Middle-Aged Americans. Washington, DC: Pew Research Center; 2013. Available from: https://www.pewresearch.org/social-trends/2013/01/30/the-sandwich-generation/. Accessed June 24, 2023.

[6] Alburez‐Gutierrez D, Mason C, Zagheni E (2021). The “sandwich generation” revisited: global demographic drivers of care time demands. Popul Dev Rev.

[7] Craig L, Mullan K (2011). How mothers and fathers share childcare: a cross-national time-use comparison. Am Sociol Rev.

[8] Evans KL, Millsteed J, Richmond JE, Falkmer M, Falkmer T, Girdler SJ (2016). Working sandwich generation women utilize strategies within and between roles to achieve role balance. PLoS One.

[9] Steiner AM, Fletcher PC (2017). Sandwich generation caregiving: a complex and dynamic role. J Adult Dev.

[10] Hämäläinen H, Tanskanen AO (2021). ‘Sandwich generation’: generational transfers towards adult children and elderly parents. J Fam Stud.

[11] Künemund H (2006). Changing welfare states and the “sandwich generation”: increasing burden for the next generation?. Int J Ageing Later Life.

[12] Polenick CA, DePasquale N, Eggebeen DJ, Zarit SH, Fingerman KL (2018). Relationship quality between older fathers and middle-aged children: associations with both parties’ subjective well-being. J Gerontol B Psychol Sci Soc Sci.

[13] Marenzi A, Pagani L (2008). The labour market participation of ‘sandwich generation’ Italian women. J Fam Econo Issues.

[14] Reczek C, Beth Thomeer M, Lodge AC, Umberson D, Underhill M (2014). Diet and exercise in parenthood: a social control perspective. J Marriage Fam.

[15] Mahne K, Huxhold O (2015). Grandparenthood and subjective well-being: moderating effects of educational level. J Gerontol B Psychol Sci Soc Sci.

[16] Marcaletti F, Cavallotti R (2021). [An exploratory study on family social capital and its relations with other forms of social capital in Spain]. Rev Esp Invest Sociol.

[17] Ghezzi S (2012). Parenthood and the structuring of time among urban households in northern Italy. Ethnol Fr.

[18] Jang SJ, Song D, Baek K, Zippay A (2021). Double child and elder care responsibilities and emotional exhaustion of an older sandwiched generation: the mediating effect of self-care. Int Soc Work.

[19] James G. IN PERSON; A Survival Course for the Sandwich Generation. The New York Times; 1999. Available from: https://www.nytimes.com/1999/01/17/nyregion/in-person-a-survival-course-for-the-sandwich-generation.html. Accessed June 24, 2023.

[20] Brenna E (2021). Should I care for my mum or for my kid? Sandwich generation and depression burden in Italy. Health Policy.

[21] Meda SG (2014). No country for old men? Italian families facing the challenges of an aging society. J Comp Fam Stud.

[22] Istat. I Tempi Della Vita Quotidiana: Lavoro Conciliazione, Parità di Genere e Benessere Soggettivo. Istat; 2019. Available from: https://www.istat.it/it/files/2019/05/ebook-I-tempi-della-vita-quotidiana.pdf. Accessed June 24, 2023.

[23] Asadzadeh M, Maher A, Jafari M, Mohammadzadeh KA, Hosseini SM (2022). A review study of the providing elderly care services in different countries. J Family Med Prim Care.

[24] Istat. Conciliazione Tra Lavoro e Famiglia. Istat; 2018. Available from: https://www.istat.it/it/files//2019/11/Report-Conciliazione-lavoro-e-famiglia.pdf. Accessed June 24, 2023.

[25] Albertini M, Tur-Sinai A, Lewin-Epstein N, Silverstein M (2022). The older sandwich generation across European welfare regimes: demographic and social considerations. Eur J Popul.

[26] Baldassar L, Wilding R, Baldock C. Long-distance care-giving: transnational families and the provision of aged care. In: Paoletti I, ed. Family Caregiving for Older Disabled People: Relational and Institutional Issues. New York, NY: Nova Science; 2010. p. 201-28.

[27] Gui T, Koropeckyj-Cox T (2016). “I am the only child of my parents:” perspectives on future elder care for parents among Chinese only-children living overseas. J Cross Cult Gerontol.

[28] Zechner M (2008). Care of older persons in transnational settings. J Aging Stud.

[29] Masten AS (2018). Resilience theory and research on children and families: past, present, and promise. J Fam Theory Rev.

[30] Pinquart M, Sörensen S (2007). Correlates of physical health of informal caregivers: a meta-analysis. J Gerontol B Psychol Sci Soc Sci.

[31] Landolfi A, Barattucci M, Lo Presti A (2020). A time-lagged examination of the Greenhaus and Allen work-family balance model. Behav Sci (Basel).

[32] Cardinali V. Dalla Fase 1 alla Fase 2: quale transizione per uomini e donne? Sintesi survey. Il lavoro di uomini e donne in tempo di Covid. Una prospettiva di genere. 2021. Available from: https://oa.inapp.org/xmlui/handle/20.500.12916/835. Accessed June 24, 2023.

[33] Rajahonka M, Villman K. Sandwich generation women in search for meaningful work and life. In: Working Women in the Sandwich Generation: Theories, Tools and Recommendations for Supporting Women’s Working Lives. Brighton, England: Emerald Publishing; 2022. p. 51-8.

[34] Cardinali V. Gender policies report 2018. Principali evidenze, spunti di riflessione. 2019. Available from: https://oa.inapp.org/xmlui/handle/20.500.12916/389. Accessed June 24, 2023.

[35] Ranji U, Frederiksen B, Salganicoff A, Long M. Women, Work, and Family During COVID-19: Findings from the KFF Women’s Health Survey. San Francisco: Kaiser Family Foundation; 2021. Available from: https://www.kff.org/womens-health-policy/issue-brief/women-work-and-family-during-covid-19-findings-from-the-kff-womens-health-survey/. Accessed August 24, 2023.

[36] Feng Z (2019). Global convergence: aging and long-term care policy challenges in the developing world. J Aging Soc Policy.

[37] Rabaglietti E, Lattke LS, Tesauri B, Settanni M, De Lorenzo A (2021). A balancing act during COVID-19: teachers’ self-efficacy, perception of stress in the distance learning experience. Front Psychol.

[38] Andruske CL, O’Connor D (2020). Family care across diverse cultures: re-envisioning using a transnational lens. J Aging Stud.

[39] Vlachantoni A, Shaw RJ, Evandrou M, Falkingham J (2015). The determinants of receiving social care in later life in England. Ageing Soc.

[40] Halperin D (2019). The intersection of formal and informal care for older people in a multicultural society: the case of two adult day-care centres in Northern Israel. Int J Care Caring.

[41] Mussida C, Patimo R (2021). Women’s family care responsibilities, employment and health: a tale of two countries. J Fam Econ Issues.

[42] İzdeş Terkoğlu Ö, Memiş E (2022). Impact of elderly care on “sandwiched-generation” women in Turkey. New Perspect Turk.

[43] Studio Associato Carniello e Vezzù. La generazione “sandwich”: i lavoratori di mezzo fra cura dei figli e familiari anziani non autosufficienti. Studio Associato Carniello e Vezzù; 2019. Available from: https://www.studiocarniello.com/la-generazione-Sandwich-i-lavoratori-di-mezzo-fra-cura-dei-figli-e-familiari-anziani-non-autosufficienti/. Accessed August 24, 2023.

[44] Sudarji S, Panggabean H, Marta RF (2022). Challenges of the sandwich generation: stress and coping strategy of the multigenerational care. Indigenous.

[45] Castro CM, King AC, Housemann R, Bacak SJ, McMullen KM, Brownson RC (2007). Rural family caregivers and health behaviors: results from an epidemiologic survey. J Aging Health.

[46] Chassin L, Macy JT, Seo DC, Presson CC, Sherman SJ (2010). The association between membership in the sandwich generation and health behaviors: a longitudinal study. J Appl Dev Psychol.

[47] Archer J, Reiboldt W, Claver M, Fay J (2021). Caregiving in quarantine: evaluating the impact of the COVID-19 pandemic on adult child informal caregivers of a parent. Gerontol Geriatr Med.

[48] Do EK, Cohen SA, Brown MJ (2014). Socioeconomic and demographic factors modify the association between informal caregiving and health in the sandwich generation. BMC Public Health.

[49] Boyczuk AM, Fletcher PC (2016). The ebbs and flows: stresses of sandwich generation caregivers. J Adult Dev.

[50] Evans KL, Millsteed J, Richmond JE, Falkmer M, Falkmer T, Girdler SJ (2019). The impact of within and between role experiences on role balance outcomes for working sandwich generation women. Scand J Occup Ther.

[51] Rari FP, Jamalludin J, Nurokhmah P (2021). Perbandingan tiPerbandingan tingkat kebahagiaan antara generasi sandwich dan non-generasi sandwichngkat kebahagiaan antara generasi sandwich dan non-generasi sandwich. Jurnal Litbang Sukowati: Media Penelitian Dan Pengembangan.

[52] Rutherford HJV, Wallace NS, Laurent HK, Mayes LC (2015). Emotion regulation in parenthood. Dev Rev.

[53] Hajal NJ, Paley B (2020). Parental emotion and emotion regulation: a critical target of study for research and intervention to promote child emotion socialization. Dev Psychol.

[54] Paley B, Hajal NJ (2022). Conceptualizing emotion regulation and coregulation as family-level phenomena. Clin Child Fam Psychol Rev.

[55] Hernandez JF (2019). The sandwich generation caregiver: hope for the future. J Clin Nurs Pract.

[56] Burke RJ. The sandwich generation: individual, family, organizational and societal challenges and opportunities. In: The Sandwich Generation. Edward Elgar Publishing; 2017.

[57] Russell BS, Tambling RR, Horton AL, Hutchison M, Tomkunas AJ (2021). Clinically significant depression among parents during the COVID-19 pandemic: examining the protective role of family relationships. Couple Family Psychol.

[58] Riley LD, Bowen CP (2005). The sandwich generation: challenges and coping strategies of multigenerational families. Fam J Alex Va.

[59] Ungar M. Social ecologies and their contribution to resilience. In: Ungar M, ed. The Social Ecology of Resilience. New York, NY: Springer; 2012. p. 13-31. 10.1007/978-1-4614-0586-3_2

[60] Turgeman-Lupo K, Toker S, Ben-Avi N, Shenhar-Tsarfaty S (2020). The depressive price of being a sandwich-generation caregiver: can organizations and managers help?. Eur J Work Organ Psychol.

[61] Greaves CE, Parker SL, Zacher H, Jimmieson NL. Resource effects in the caregiving process. In: Burke RJ, Calvano LM, eds. The Sandwich Generation: Caring for Oneself and Others at Home and at Work. Cheltenham, England: Edward Elgar Publishing; 2017. p. 99-125.

[62] Erlandsson LK (2013). The redesigning daily occupations (ReDO)-program: supporting women with stress-related disorders to return to work—knowledge base, structure, and content. Occup Ther Ment Health.

[63] Johansson G, Eklund M, Erlandsson LK (2012). Everyday hassles and uplifts among women on long-term sick-leave due to stress-related disorders. Scand J Occup Ther.

[64] Tebes JK, Irish JT (2000). Promoting resilience among children of sandwiched generation caregiving women through caregiver mutual help. J Prev Interv Community.

[65] Seshachalam PA (2016). Healthy way to handle work place stress through yoga, meditation and soothing humor. IOSR J Bus Manage.

